# Derotational distal femur osteotomy with medial patellofemoral ligament reconstruction can get good outcomes in the treatment of recurrent patellar dislocation with excessive TT-TG and increased femoral anteversion

**DOI:** 10.3389/fsurg.2024.1392947

**Published:** 2024-04-10

**Authors:** Zhenhui Huo, Kuo Hao, Chongyi Fan, Yingzhen Niu, Haotian Bai, Weixia Bai

**Affiliations:** ^1^ Department of Orthopaedic Surgery, Third Hospital of Hebei Medical University, Shijiazhuang, Hebei, China; ^2^ School of Basic Medical Science, Hebei University, Baoding, Hebei, China

**Keywords:** patellar dislocation, derotational distal femur osteotomy, tibial tubercle osteotomy, femoral anteversion angle, tibial tubercle trochlear groove distance, patellofemoral congruence, medial patellofemoral ligament reconstruction

## Abstract

**Background:**

Surgery is the main treatment for recurrent patellar dislocation (PD). However, due to the complexity of anatomical factors, there is still a lack of consensus on the choice of combined surgical methods. This study aimed to compare the clinical and radiological outcomes of medial patellofemoral ligament reconstruction combined with derotational distal femur osteotomies (MPFLR + DDFO) and combined with tibial tubercle osteotomies (MPFLR + TTO) for recurrent PD with increased femoral anteversion angles (FAA) and excessive tibial tubercle-trochlear groove (TT-TG) distance.

**Methods:**

In this retrospective analysis, MPFLR + DDFO and MPFLR + TTO patients from 2015 to 2020 were included. Group A (MPFLR + DDFO, *n* = 42) and B (MPFLR + TTO, *n* = 46) were formed. Clinical outcomes included physical examinations, functional outcomes (Kujala, Lysholm, International Knee Documentation Committee (IKDC), visual analog scale (VAS) and intermittent and persistent osteoarthritis pain scale (ICOAP), Tegner scores), and complications. The Caton-Deschamps index (CD-I), patellar title angle, patellar congruence angle, patella-trochlear groove distance, TT-TG distance, and FAA were used to assess radiological outcomes.

**Results:**

All clinical outcomes improved significantly in both groups, but Group A had significantly better postoperative scores than Group B (Kujala: 89.8 ± 6.4 vs. 82.9 ± 7.4, *P* < 0.01; Lysholm: 90.9 ± 5.1 vs. 81.3 ± 6.3, *P* = 0.02; IKDC: 87.3 ± 9.0 vs. 82.7 ± 8.0, *P* < 0.01; Tegner: 6.0 (5.0, 9.0) vs. 5.0 (4.0, 8.0), *P* = 0.01). However, there was no significant difference in the VAS and ICOAP scores between the two groups. No dislocation recurrences occurred. Radiological outcomes improved significantly in both groups, but Group A had better outcomes. After surgery, the patellar height of 88.5% (23/26) patients in Group A and 82.8% (24/29) patients in Group B was restored to normal (the Caton-Deschamps index <1.2).

**Conclusions:**

Both MPFLR + TTO and MPFLR + DDFO obtained satisfactory clinical and radiological outcomes in the treatment of recurrent PD with increased FAA and excessive TT-TG. However, the outcomes of MPFLR + DDFO were better and should be considered a priority. MPFLR + TTO may be not necessary for such patients.

## Introduction

Patellar dislocation (PD) is a complex, multifaceted condition that has a high incidence in adolescents (1, 2). PD occurs more frequently in the second decade of life, mainly in female adolescent patients. The incidence of PD varies between 5.8/100,000 and 29/100,000 in the 10–17-year-old age group (3, 4). PD can lead to adolescent patellofemoral symptoms and impact the adolescent's mental health (5).

The patellofemoral joint stability depends on both bone morphology and soft tissue restraints, and it has a role of fundamental importance for proper functioning of the knee extensor mechanism (4). Abnormal pathology and anatomy of soft tissues and bones are present in PD, including disruption of medial soft tissue, trochlear dysplasia, patella alta, torsional deformity, and excessive tibial tubercle-trochlear groove (TT-TG) distance (6, 7). Among them, excessive TT-TG and increased femoral anteversion (FA), as important risk factors for PD, have attracted more and more attention in recent years (6, 8–10).

Increased FA could result in a more mismatched position of the patella relative to the femoral trochlea, increasing patella outward-migration and pressure on the lateral articular surface, leading to PD (11, 12). The TT-TG distance represents the radiographic measurement of the lateral quadriceps vector acting on the patella. The lateralized tibial tubercle, which can be measured by TT-TG distance, leads to the increase in *Q* angle and a lateral force on the patella, thus damaging the normal patellar tracking (4, 13, 14).

For different risk factors of PD, Dejour et al. proposed the concept of “menu à la carte” (15, 16), that is, according to the different anatomical abnormalities of patients, different surgical combinations should be selected to achieve “suit the remedy to the case”. Medial patellofemoral ligament reconstruction (MPFLR) is a basic procedure, which leads to good outcomes in various clinical studies (17). Tibial tubercle osteotomy (TTO) is a conventional surgical plan for the treatment of excessive TT-TG distance. Therefore, MPFLR combined with TTO has become a common treatment strategy for patients with recurrent PD and excessive TT-TG distance. But no single procedure can be applicable in all cases considering multifactorial etiology (18), most of these studies didn't discuss the impact of increased FA. Zhang et al. reported that the increased FA angle (FAA) was one of the reasons for the failure of MPFLR combined with TTO (7). As a surgical indication for PD patients with FAA > 25° (19–21), derotational distal femoral osteotomy (DDFO) can improve the relationship between the patella and the trochlea and increase the lateral resistance of the patella during flexion and extension, thereby treating PD (9). Besides, some studies showed that the TT-TG distance decreased after DDFO (22). Therefore, for patients with increased FAA and excessive TT-TG distance, whether MPFLR combined with DDFO is sufficient to achieve favorable results, or whether additional TTO is required is still controversial.

At present, there is no research comparing the long-term efficacy of MPFLR combined with DDFO and MPFLR combined with TTO (MPFLR + DDFO vs. MPFLR + TTO) in the treatment of recurrent PD with increased FAA and excessive TT-TG distance. The present study aimed to evaluate the clinical and radiological parameters of MPFLR + DDFO and MPFLR + TTO in patients having both increased FAA and excessive TT-TG distance. It was hypothesized that MPFLR + DDFO would lead to better postoperative patellar stability and patient-reported outcomes than MPFLR + TTO.

## Materials and methods

### Patient selection

Before the study began, approval was obtained from the hospital's ethics committee. Informed consent was obtained from all participants.

The researchers reviewed medical records for patients with recurrent PD, having both increased FAA and excessive TT-TG distance, who underwent MPFLR + DDFO or MPFLR + TTO were enrolled in this retrospective study, between 1 January 2015 and 31 August 2020. The recurrent PD was diagnosed based on the medical history, physical examination, and imaging evaluation. The inclusion criteria were as follows: (1) two or more episodes of lateral patellar dislocation; (2) FAA > 25° and TT-TG distance >20 mm; (3) closed epiphyseal growth plates; (4) no history of previous surgery on the injured knee; and (5) at least 36 months of follow-up, and (6) complete medical records (including imaging data at postoperative follow-up).

The exclusion criteria were as follows: (1) acute, traumatic or habitual dislocation; (2) lower limb malalignment (>5° varus or valgus); (3) concomitant ligament reconstruction (cruciate ligament or collateral ligament); (4) high-grade trochlear dysplasia [grades B, C or D of Dejour's classification (23)]; and (5) generalized or localized joint laxity. To ensure a rigorous selection process, the enrolled participants were rechecked independently and confirmed by the same senior orthopedist, who has extensive experience in treating patellofemoral disorders.

Between 2015 and 2017, patients with FAA > 25° and TT-TG distance >20 mm were treated with MPFLR + TTO. After 2017, such patients were treated with MPFLR + DDFO if no other bony deformities existed.

**Figure 1:**
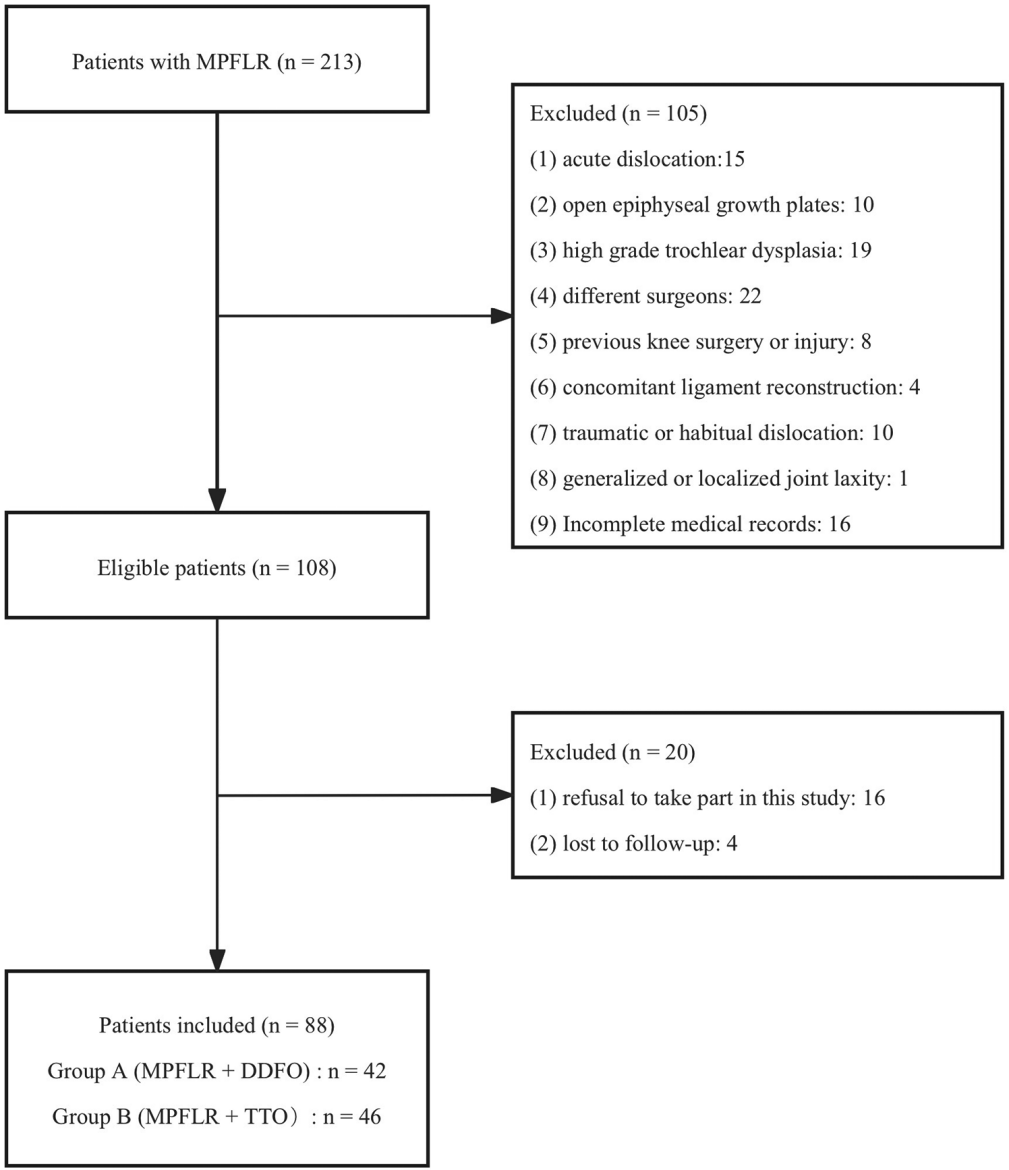
The flowchart of the patient selection. MPFLR + DDFO and MPFLR + TTO represent MPFLR combined with DDFO and MPFLR combined with TTO, respectively. MPFLR, medial patellofemoral ligament reconstruction; DDFO, derotational distal femoral osteotomy; TTO, tibial tubercle osteotomy.

Based on the inclusion and exclusion criteria, 104 patients (104 knees) were included in this study. Patients were allocated into two groups according to whether DDFO or TTO was performed in addition to MPFLR. 53 patients (53 knees) underwent MPFLR + DDFO (Group A), and 51 patients (51 knees) underwent MPFLR + TTO (Group B) (Figure 1). All surgeries were performed by the same senior surgeon with more than 20 years of experience. All radiological evaluations were performed on computer tomography (CT) images within 1 week before surgery and at 1 year after surgery. And weight-bearing anteroposterior and lateral radiographs were taken at each follow-up after surgery. All clinical evaluations were performed before and 3 years after surgery. Basic demographic and clinical data were collected from the medical records, including age, sex, body mass index (BMI), and intraoperative blood loss.

### Evaluation methods

#### Clinical evaluation

Physical examination included an apprehension test, the range of motion (ROM) and patellar tracking indicated by J-sign. The clinical outcomes were performed including the Kujala score, Lysholm score, International Knee Documentation Committee (IKDC) score, Tegner activity score, visual analog scale for pain (VAS) score and intermittent and persistent osteoarthritis pain scale (ICOAP) score (24–27). In this study, the improvement of knee function was evaluated by the changes of knee function scores (such as the Kujala score, Lysholm score, IKDC score and Tegner activity score). The VAS and ICOAP scores were used to assess knee pain. Demographic factors and patient records with a special focus on postoperative PD recurrence and complications were analyzed. A poor outcome was identified as patellar subluxation or dislocation recurrence, subjective instability and complications. The postoperative follow-up work was performed independently by two blinded surgeons.

#### Radiological evaluation

**Figure 2:**
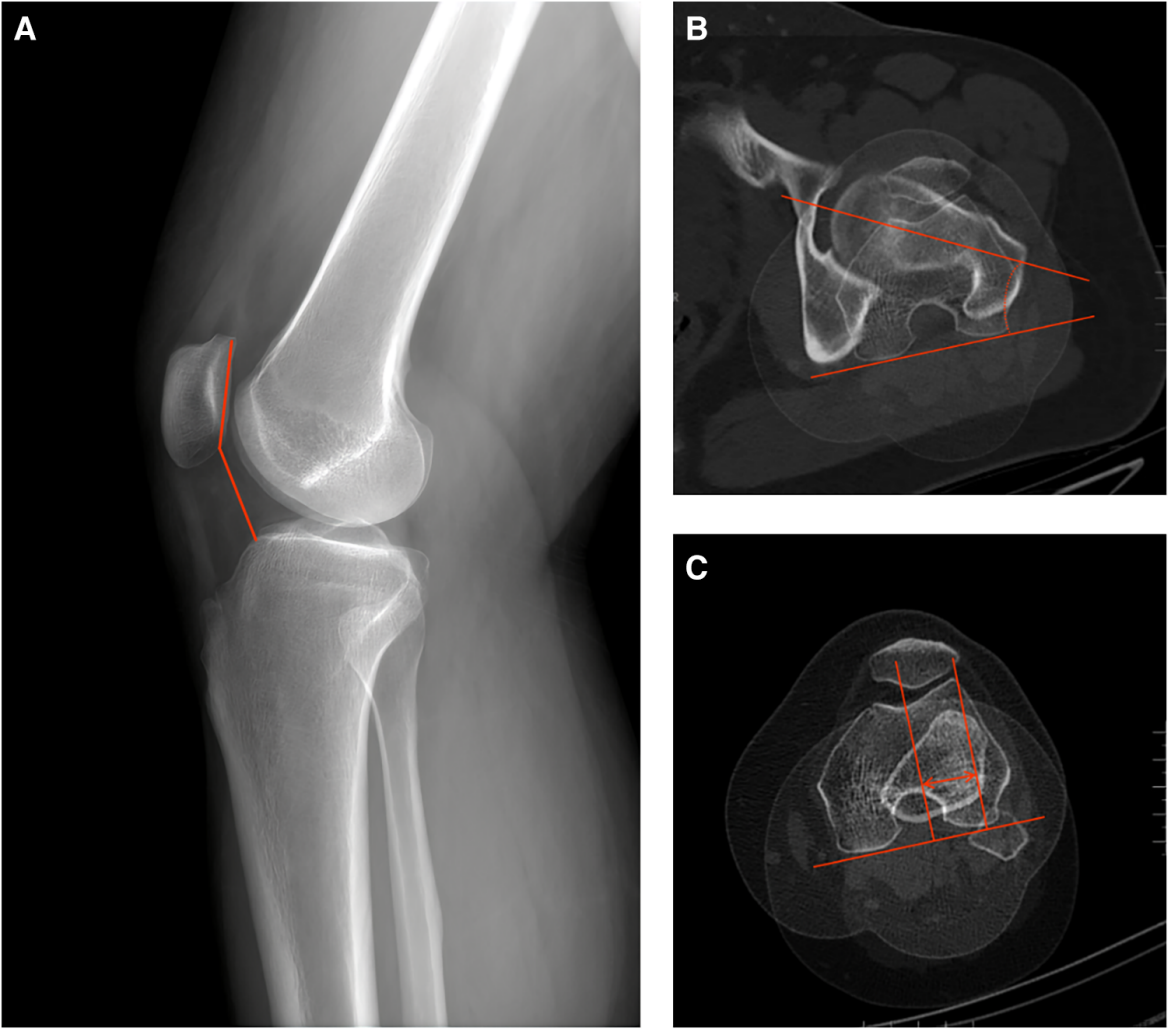
The schematic diagrams of measurement of CD-I, TT-TG distance, and FAA. (**A**) CD-I: the ratio of the distance from the lowest point of the patellar articular surface to the upper corner of the tibial plateau to the length of the patellar articular surface. (**B**) FAA: the angle between the axis of the femoral neck connecting the center of the femoral head and the midpoint of the femoral neck, and the posterior condylar line connecting the most posterior points of the medial and lateral femoral condyles. (**C**) TT-TG distance: the distance between two lines drawn from the deepest point of the trochlear groove and the center of the tibial tuberosity, respectively, perpendicular to the posterior condylar line. CD-I, caton-deschamps index; TT-TG, tibial tuberosity-trochlear groove; FAA, femoral anteversion angle.

The CD-I was defined as the ratio of the distance from the lowest point of the patellar articular surface to the proximal anterior edge of the tibia to the length of the patellar articular surface (patella alta: CD-I > 1.2) (Figure 2A). The FAA was defined as the angle between the axis of the femoral neck connecting the center of the femoral head and the midpoint of the femoral neck, and the posterior condylar line (PCL) connecting the most posterior points of the medial and lateral femoral condyles on the axial slice showing the posterior condyles with the Roman arch (28) (Figure 2B). The TT-TG distance was measured on two overlapped axial CT images, including the deepest point of the trochlear groove and approximately one-third of the proximal tibial tuberosity. The distance between two lines drawn from the deepest point of the trochlear groove and the center of the tibial tuberosity, respectively, perpendicular to the PCL, was measured as the TT-TG distance (29) (Figure 2C).

**Figure 3:**
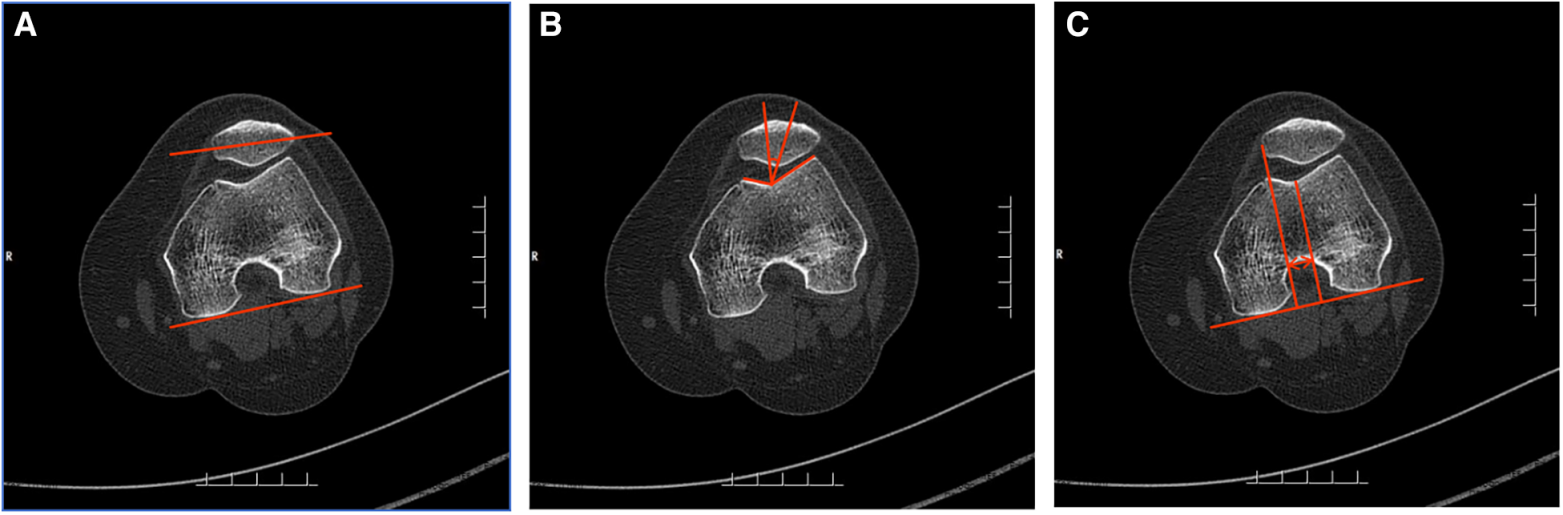
The schematic diagrams of measurement of patellofemoral congruence. (**A**) PTA: the angle between the line connecting the posterior of the medial and lateral condyles of the femur and the line extending the maximum transverse diameter of the patella. (**B**) PCA: the angle between the line bisecting sulcus angle and the line connecting the deepest point of the sulcus and the lowest point of the patella. (**C**) Patella–trochlear groove distance: the distance between the perpendicular line of the posterior condylar line through the deepest point of the trochlear groove and the medial edge of the patella. PTA, patella tilt angle; PCA, patellar congruence angle.

Patellofemoral congruence was indicated by the patellar tilt angle (PTA), patellar congruence angle (PCA) and patella-trochlear groove (PTG) distance. PTA, PCA and PTG distance were measured as previously described by Hao et al. (30) (Figure 3).

All radiological evaluations were performed independently by two experienced surgeons twice using the same criteria at a 2-week interval, and the means of the data were taken as the final results for analysis.

### Surgical technique

#### *Medial patellofemoral ligament reconstruction* (in both groups the same)

**Figure 4:**
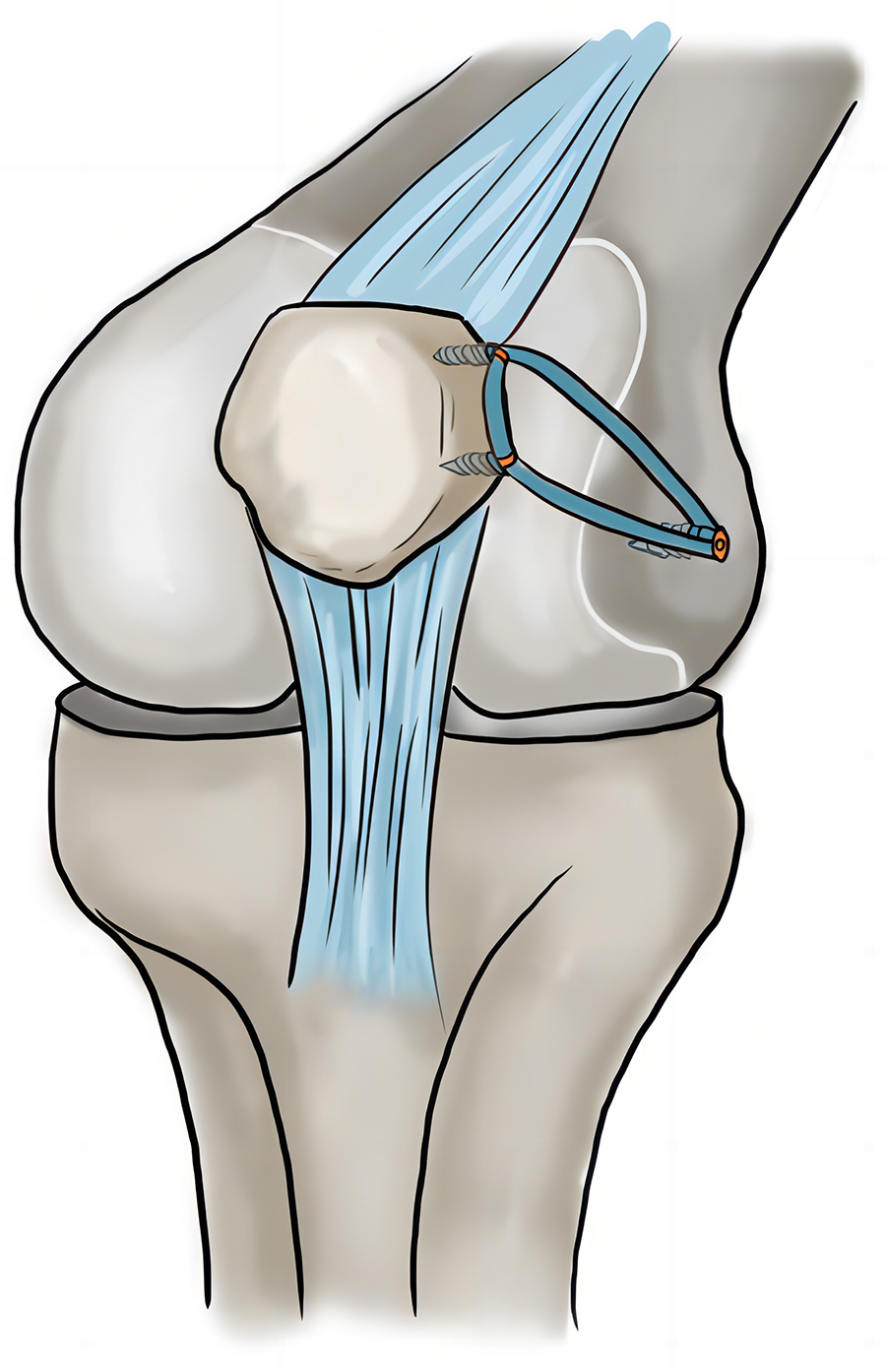
The schematic diagram of MPFLR. MPFLR, medial patellofemoral ligament reconstruction.

After successful anesthesia, the patient was placed in the supine position with a tourniquet tied to the proximal thigh for the duration of the operation to facilitate visualization. The pressure was 2 times of the patient's systolic blood pressure, and the time was 90 min. The tourniquet was used after the lower limbs were drained. A 2-cm skin incision was made on the proximal medial tibia to separate and expose the “pes anserinus”. The autologous semitendinosus was cut and folded after repair. The tendons were knitted and sewed with the irretrievable thread of Ai-Bang No.1 to reserve 2 cm at the back end. The femoral tunnel was located at the midpoint of the adductor tubercle and medial epicondyle of the femur. The fixation method on the patellar side was to use 2 suture anchors. The femoral side is fixed with an absorbable screw. Intraoperative fluoroscopy was used to determine the femoral insertion point by the Schottle method. After fixation, the patellar tracking, graft tension, and the lateral retinacula tightness were checked by arthroscopy in extension and flexion. If the patella was stable, the femoral end would be fixed (Figure 4). The tourniquet was loosened after the combined surgery (MPFLR + DDFO or MPFLR + TTO).

#### Derotational distal femur osteotomy

**Figure 5:**
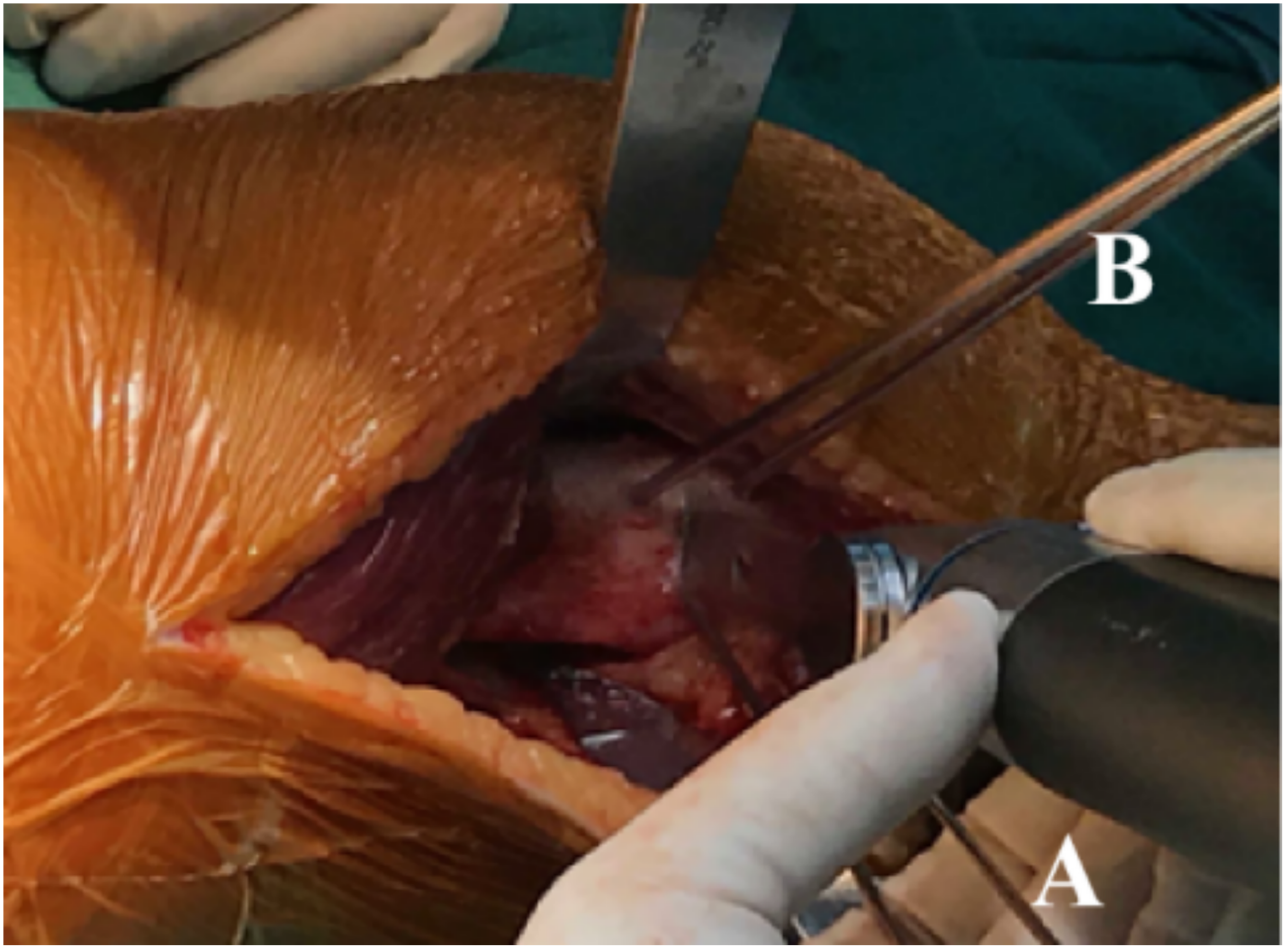
An intraoperative photograph of the derotational distal femoral osteotomy. (**A**) The osteotomy level was determined by two K wires. (**B**) The derotational angle was controlled by another two K wires.

**Figure 6:**
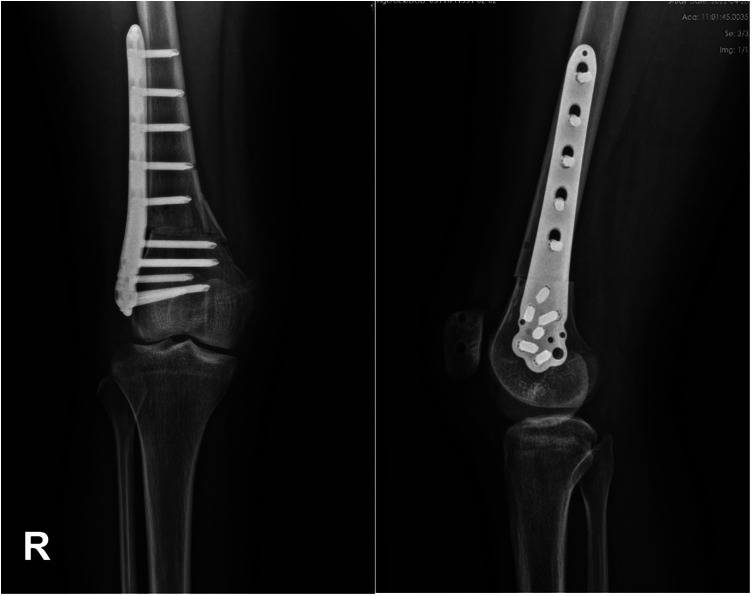
Radiograph and CT scan after MPFLR combined with DDFO. MPFLR, medial patellofemoral ligament reconstruction; DDFO, derotational distal femur osteotomy.

The target postoperative FAA value was 10°. DDFO was performed with a 10-cm incision on the lateral thigh along the longitude axis of the distal femur. The distal femoral shaft was exposed through the intermuscular space after the separation of the subcutaneous tissue and incision of the borders of the biceps femoris and vastus lateralis. The supracondylar osteotomy line was marked with two Kirschner wires, which were set parallel to the tibiofemoral joint line. Then the Kirschner wires were inserted into the osteotomy line to determine the rotational angle between the two wires based on the preoperative CT measurements, which was verified by intraoperative fluoroscopy. After osteotomy with an oscillating saw according to the direction of the guide pin, the distal femur was externally rotated to a predetermined angle to adjust the rotational alignment and correct increased FAA. After temporarily fixing the femoral shaft, fluoroscopy was used to check the reduction of the femoral shaft. Finally, the distal femur was fixed with one lateral femur plate (Figure 5). The femur was fixed using a Tomofix distal femoral plate (DePuy Synthes, Umkirch, Germany) with 9 screws after osteotomy (Figure 6). Stop bleeding and suture the wound layer by layer.

#### Tibial tubercle transfer (medialization tubercle osteotomy)

**Figure 7:**
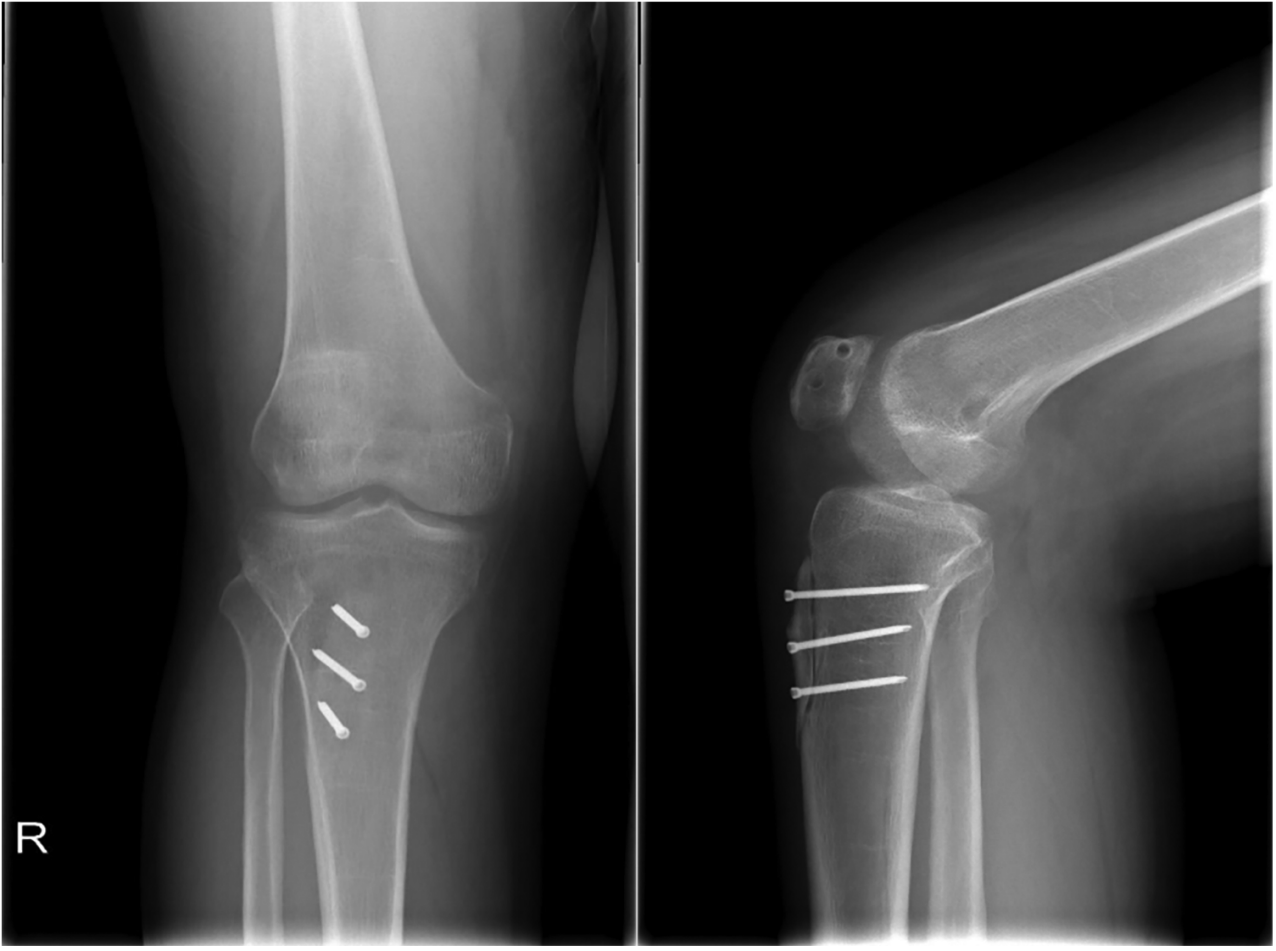
Radiograph after MPFLR combined with TTO. MPFLR, medial patellofemoral ligament reconstruction; TTO, tibial tubercle osteotomy.

Osteotomies usually started at the patellar tendon-bone junction at the proximal tibial tubercle, and the transverse osteotomy was performed. The distance of intraoperative displacement was determined using CT measurement before the operation to recover the TT-TG to 10–12 mm. The midpoint of the osteotomy area was taken as the measuring point, and the distance of internal displacement was determined with a measuring ruler. The tubercle was moved medially and temporarily fixed with two Kirschner wires. The patellar motion was measured by the flexion and extension of the knee joint. The position of the patella was observed by fluoroscopy of the knee at 30° to avoid overcorrection. The tibial tubercle was fixed firmly with cortical nails or screws (Figure 7). Stop bleeding and suture the wound layer by layer.

### Postoperative rehabilitation

Both groups of patients were provided with rehabilitation guidance and training by the integrated team of medical care and rehabilitation under the same overall responsibility system nursing. Postoperative enhanced recovery after surgical nursing was carried out in patients with postoperative education, wound nursing, functional exercise, pain management, dietary intervention, and psychological nursing. The aim is to accelerate the postoperative recovery process of patients, reduce the pain degree of postoperative incision and improve the knee joint function of patients by optimizing the postoperative nursing of patients. A protective knee brace was applied during the first 6 weeks after surgery. On the first day after surgery, quadriceps femoris muscle strength recovery training and patella shift training were performed, and progressive knee motion training was performed. The range of motion (ROM) was 0°–45° within 2 weeks, knee flexion was 90° within 4 weeks, and ROM was gradually increased to 110° within 6 weeks. After 8 weeks, ROM was unrestricted and weight-bearing activity was authorized. Rehabilitation exercise should include straight-leg-raising. Patients who achieved full ROM and normal muscle strength and stability were allowed to return to normal sports activities at 6 months. Strenuous high-risk exercise required lengthier recuperation depending on the individual.

### Statistical analysis

All statistics were conducted using SPSS software Version 26.0 (IBM Corp). Means and standard deviations were used to describe continuous variables, and proportions were used to describe categorical variables. The Shapiro–Wilk test was used to determine the normality of the data. Student-*t*-test and *χ*^2^ test were used to compare the difference between two groups. The matched *t*-test was used to calculate differences between preoperative and postoperative. *P* < 0.05 indicated a statistically significant difference. The interobserver and intraobserver agreement of the measurements was evaluated with intraclass correlation coefficients (ICCs). ICC > 0.8 was considered excellent. It was shown that the included sample size could achieve an adequate power of >0.99 with an alpha of 0.05 (G*Power 3.1.97, Düsseldorf, Germany) (31).

## Results

A total of 88 patients (88 knees) were included in this study, and the follow-up time was 39.6 ± 3.7 months (range 36–43 months). The patient demographics and clinical data were shown in Table 1, and no significant difference was observed between the two groups. The inter- and intra-observer reliability of the measurement was found for all measurements, with an ICC of >0.8 (Table 2). All quantitative data in this study passed the test of normality.

**Table 1:** Demographic characteristics of patients.

	Group A	Group B	*P*-value
Number of patients	53	51	–
Number of knees	53	51	–
Side (left/right)	26/27	24/26	n.s
Sex (male/female)	19/34	22/29	n.s
Age (years)	22.3 ± 4.1	23.1 ± 4.9	n.s
BMI (kg/m^2^)	21.6 ± 1.5	20.8 ± 2.1	n.s
Follow-up (months)	39.6 ± 3.1	40.1 ± 3.9	n.s
Intraoperative blood loss (ml)	60.7 ± 25.9	55.9 ± 22.5	n.s

Data are presented as mean ± standard deviation or number.

BMI, body mass index; n.s, non-significant.

**Table 2:** The inter- and intra-observer reliability of different measurements.

	Interobserver reliability	Intraobserver reliability
FAA	0.874 (0.812–0.914)	0.923 (0.872–0.946)
TT-TG distance	0.925 (0.872–0.943)	0.891 (0.862–0.910)
PTA	0.899 (0.846–0.921)	0.904 (0.872–0.932)
CD-I	0.910 (0.811–0.922)	0.889 (0.879–0.936)
PCA	0.882 (0.837–0.926)	0.914 (0.865–0.948)
PTG distance	0.917 (0.901–0.968)	0.874 (0.874–0.937)

FAA, femoral anteversion angle; TT-TG, tibial tuberosity-trochlear groove; CD-I, caton-deschamps index; PTA, patellar tilt angle; PCA, patellar congruence angle; PTG, patella-trochlear groove.

### Clinical outcomes

From preoperative to postoperative, all knee function outcomes (Kujala score, Lysholm score, IKDC score, Tegner activity score, VAS score and ICOAP score) of Group A and Group B were significantly improved. At the last follow-up, there was significant difference in Kujala score, Lysholm score, IKDC score and Tegner activity score between the two groups, and Group A had higher scores than Group B (Table 3). However, although VAS and ICOAP scores as indicators of pain were significantly improved after surgery, there was no difference between the two groups.

**Table 3:** Comparison of clinical outcomes.

	Group A	Group B	*P*-value
Kujala score			
Preoperative	41.9 ± 7.9	39.2 ± 6.1	n.s
Postoperative	87.3 ± 6.2	80.7 ± 7.4	<0.01
*P*-value	<0.01	<0.01	
Lysholm score			
Preoperative	39.4 ± 8.0	36.9 ± 5.1	n.s
Postoperative	90.8 ± 5.1	81.2 ± 6.3	0.02
*P*-value	<0.01	<0.01	
IKDC score			
Preoperative	48.7 ± 9.5	47.8 ± 9.3	n.s
Postoperative	87.2 ± 10.0	81.7 ± 8.1	<0.01
*P*-value	<0.01	<0.01	
Tegner activity score			
Preoperative	3.0 (3.0, 5.0)	3.0 (3.0, 6.0)	n.s
Postoperative	6.0 (5.0, 9.0)	5.0 (4.0, 8.0)	0.01
*P-*value	<0.01	<0.01	
VAS score			
Preoperative	7.8 ± 1.5	7.7 ± 1.1	n.s
Postoperative	1.5 ± 0.4	1.5 ± 15.2	n.s
*P*-value	<0.01	<0.01	
ICOAP score			
Preoperative	65.5 ± 9.0	66.0 ± 8.6	n.s
Postoperative	14.6 ± 3.9	16.6 ± 5.0	n.s
*P*-value	<0.01	<0.01	

Data are presented as mean ± standard deviation unless otherwise indicated.

IKDC, international knee documentation committee; VAS, visual analog scale for pain; ICOAP, intermittent and persistent osteoarthritis pain scale; n.s, non-significant.

The apprehension sign, J-sign and knee ROM were shown in Table 4. Both the apprehension sign and J-sign were significantly improved in both groups after surgery. At the last follow-up, it was found that the apprehension sign and residual J-sign in Group A were less than those in Group B, and the difference was statistically significant. However, there was no significant difference in the ROM between the preoperative and postoperative follow-up and between the two groups.

**Table 4:** The apprehension sign, J-sign and the range of motion in group A and group B.

	Group A	Group B	*P*-value
Apprehension sign			
Preoperative	41 (77.4%)	40 (78.4%)	n.s
Postoperative	1 (1.9%)	6 (11.8%)	<0.01
*P*-value	<0.01	<0.01	
J-sign			
Preoperative	39 (73.6%)	37 (72.5%)	n.s
Postoperative	5 (9.4%)	9 (17.6%)	0.02
*P*-value	<0.01	<0.01	
Passive ROM knee flexion (deg)			
Preoperative	135.2 (130.0–137.0)	134.9 (131.0–135.0)	n.s
Postoperative	135.1 (129.0–138.0)	134.1 (128.0–137.0)	n.s
*P*-value	n.s	n.s	
Passive ROM knee extension (deg)			
Preoperative	1.7 (0.0–3.0)	1.9 (0.0–3.0)	n.s
Postoperative	1.4 (0.0–4.0)	1.8 (0.0–4.0)	n.s
*P*-value	n.s	n.s	

Data are presented as mean ± standard deviation or number (proportion).

ROM, range of motion; n.s, non-significant.

### Radiographic outcomes

As is shown in Table 5, radiological outcomes improved significantly in both groups, but Group A showed better outcomes. The mean FAA in Group A was corrected from 32.3 ± 4.3° to 12.3 ± 3.1° (*P* < 0.01). The change of FAA in Group B between pre- and postoperatively was no significant difference. The mean TT-TG in Group A was corrected from 21.5 ± 3.5 to 17.6 ± 2.7 (*P* < 0.01). The mean TT-TG in Group B was corrected from 23.1 ± 4.1 to 13.0 ± 2.2 (*P* < 0.01). There were 26 and 39 patients with patella alta (CD-I > 1.2) before surgery in Group A and Group B, respectively. After MPFLR + DDFO and MPFLR + TTO, the patellar height of 88.5% (23/26) patients in Group A and 82.8% (24/29) patients in Group B restored to normal (CD-I < 1.2).

**Table 5:** Comparison of radiographic outcomes.

	Group A	Group B	*P*-value
FAA (deg)			
Preoperative	32.4 ± 4.6	31.5 ± 5.1	n.s
Postoperative	12.5 ± 3.3	31.5 ± 5.0	<0.01
*P*-value	<0.01	n.s	
TT-TG distance (mm)			
Preoperative	21.4 ± 3.5	22.1 ± 4.3	n.s
Postoperative	15.1 ± 2.3	10.2 ± 1.6	<0.01
*P*-value	<0.01	<0.01	
CD-I			
Preoperative	1.16 ± 0.09	1.14 ± 0.12	n.s
Postoperative	0.91 ± 0.08	0.98 ± 0.13	<0.01
*P*-value	<0.01	<0.01	
PTA (deg)			
Preoperative	27.4 ± 3.8	26.8 ± 2.4	n.s
Postoperative	12.4 ± 2.8	14.6 ± 3.7	<0.01
*P*-value	<0.01	<0.01	
PCA (deg)			
Preoperative	41.3 ± 5.4	42.8 ± 5.4	n.s
Postoperative	15.8 ± 5.9	18.7 ± 6.4	0.03
*P*-value	<0.01	<0.01	
PTG distance (mm)			
Preoperative	20.4 ± 7.4	21.8 ± 7.5	n.s
Postoperative	8.3 ± 2.2	10.1 ± 2.9	0.01
*P*-value	<0.01	<0.01	

Data are presented as mean ± standard deviation.

FAA, femoral anteversion angles; TT-TG, tibial tubercle trochlear groove; CD-I, caton-deschamps index; PTA, patellar tilt angle; PCA, patellar congruence angle; PTG, patella-trochlear groove; n.s, non-significant.

The PTA, PCA, and PTG distance demonstrated significant improvements in both groups (*P* < 0.01), while Group A had better outcomes than Group B.

### Complications

No patients with patellar subluxation or dislocation recurrence were observed. The imaging data at three months after surgery showed that 87.0% (40/46) patients in Group B achieved healing of the osteotomy, and 85.7% (35/42) patients in Group A healed. The proportion of bone healing in Group B was higher, but there was no significant difference. At 6 months after operation, all patients achieved bone healing of the osteotomy. Complications such as deep venous thrombosis of the lower extremities, postoperative knee stiffness and soreness and swelling weren't found in both groups. Two patients in Group A had postoperative limb weakness. However, in Group B, limb weakness was observed in five patients. One patient had wound infection after operation. Three patients underwent hardware removal due to postoperative pain (Table 6).

**Table 6:** The primary postoperative complication in group A and group B.

	Group A	Group B
Wound infection	0	1 (2.0%)
Deep vein thrombosis	0	0
Unplanned removal due to pain	0	3 (6.0%)
Weakness	2 (3.8%)	5 (9.8%)
Soreness and swelling	0	0
Joint stiffness	0	0
Patellofemoral osteoarthritis	0	0

Data are presented as number (percentage).

## Discussion

The most important finding in the study was that patients having both increased FAA and excessive TT-TG distance who underwent MPFLR + DDFO had better clinical and radiological outcomes than those who underwent MPFLR + TTO during a minimum of 36 months of follow-up. MPFLR + TTO may not be a priority for such patients.

The treatment of patients with PD remains a great challenge for surgeons. MPFLR has become the main surgical intervention for patients with failed conservative treatment in recent years because of its less surgical trauma, favorable clinical outcomes, and low PD recurrence (32, 33). MPFLR is considered the gold standard in the treatment of recurrent PD, achieving excellent results with a reported risk for recurrent instability of less than 2% (34). Sappey-Marinier et al. reported a satisfactory clinical outcome of 211 patients who underwent isolated MPFLR regardless of bony deformities (35). Recently, another study supported the view that the isolated MPFLR could be effective in treating RPD with bony risk factors, including increased FAA (36). Although MPFLR alone has significant improvements in clinical and radiological parameters, it is not as good as MPFLR combined with other combined operations in some selected patients (12, 30).

FAA of normal adults is between 5° and 15°. FAA > 25° or 30° is reported as one of the risk factors for malalignment between the patella and trochlea, because it generates increased tension of the medial patellofemoral ligament (23, 37), resulting in abnormal patellofemoral loads and the tendency for lateral dislocation (38). Besides, increased FAA can cause various clinical manifestations, including anterior knee pain, patellar dislocations or subluxations, and abnormal gait (39). A cadaver study also showed that isolated MPFLR was insufficient to restore patellar kinematics and patellofemoral pressure with increased FAA (40). This may be because the continuous unilateral force vector still acts on the patella, which may increase the relaxation of the graft and the pressure on the reconstructed MPFL, resulting in graft failure and PD recurrence (12). As a mature surgical protocol, DDFO can improve increased FAA and the relationship between the shaft of the femoral neck and the coronal plane of the femoral condyles and recover patella tracking (41).

Some studies demonstrated that TT-TG distance was correlated with gender, size of femur torsion, and tibial torsion (42). Up to now, as a surgical scheme to solve the problem of excessive TT-TG distance, TTO has been controversial, whether it is performed alone or combined with other operations. Isolated MPFLR in PD presents a functional improvement, with a low rate of complications and failure, regardless of the pre surgical TT-TG (8, 43). On the contrary, Stephen et al. and Franciozi et al. have found that MPFLR + TTO had better clinical effect and patellofemoral movement than MPFLR alone (44, 45). In the study of Ahmad R et al., the complications of TTO cannot be ignored (17). In addition, some studies showed that TT-TG distance decreased after DDFO (22, 46, 47). In this study, the TT-TG distance of patients decreased from 21.5 ± 3.5 to 17.6 ± 2.7 postoperation, and as of the last follow-up, there were no patients with secondary dislocation, which indicated that TTO may be not necessary for patients having both increased FAA and excessive TT-TG who receive MPFLR + DDFO.

MPFLR + DDFO is a good procedure for patients with PD with increased FAA, and good clinical outcomes can be obtained in many studies (12, 40). Hao et al. reported that MPFLR + DDFO or MPFLR alone could yield satisfactory clinical and radiological outcomes, but better results were obtained with MPFLR + DDFO for the patients with increased FAA (30). Tian et al. reported that combined surgery can achieve good clinical results, including improving TT-TG distance and PTA, and improving knee joint function (46). Zhang et al. reported that Kujala, IKDC, and Lysholm scores were significantly improved without complications (12). These results were also remarkable when the patients had a preoperative high-grade J-sign (39). The studies conducted by Franciozi et al. and Zhang et al. have both found a negative correlation between increased FAA and clinical outcomes following MPFLR and combined TTO (7, 48). This also confirms that MPFLR + DDFO is a better choice than MPFLR + TTO when FA increases.

However, few studies have directly compared the clinical and radiological parameters of MPFLR + DDFO and MPFLR + TTO in the treatment of recurrent PD patients having both increased FAA and excessive TT-TG distance. The results showed that for recurrent PD patients having excessive TT-TG distance and increased FAA, MPFLR + DDFO was superior to MPFLR + TTO in both clinical and radiological outcomes. During at least 36 months follow-up time, no PD recurrence was observed in both groups. The patellofemoral fitness of the two groups was also significantly improved. And both groups achieved significant improvements in function outcomes, including knee function, pain relief, patellar tracking, and patellofemoral stability. However, there were significant differences between MPFLR + DDFO and MPFLR + TTO, indicating that although the osteotomy line of Group A was larger than that of Group B, the healing of the osteotomy line would not pose a greater potential risk to the recovery of knee function. The activity level assessed by the Tegner activity score was higher in Group A, which is important for improving quality of life, as patients with PD are mostly young active children and adolescents. The TT-TG distance was improved in both groups. In group A, the external rotation of the trochlear groove caused by DDFO may improve the excessive TT-TG distance.

Interestingly, the height of the patella decreased after surgery. After MPFLR + DDFO and MPFLR + TTO, the patellar height of 88.5% (23/26) patients in Group A and 82.8% (24/29) patients in Group B restored to normal (CD-I < 1.2). A study by Woodmass JM et al. showed similar results. In his research, isolated MPFLR resulted in a statistically significant decrease in patellar height, and the necessity of tibial tubercle distalization in patients with patella alta should be a focus of further research (49).

However, in the VAS and ICOAP pain scores, although patients in Group A had less pain than patients in Group B, there was no significant difference. And there was no difference in postoperative ROM. It may be that both surgical methods are relatively large operations, which usually require relatively long braking and recovery time. In the short-term follow-up, the difference may not be significant.

In this study, we observed that patients in Group B had multiple complications. Based on previous studies, multiple complications were reported, including trochanteric fractures, delayed union, need to remove internal fixation, deep vein thrombosis, and joint stiffness (50, 51). In this study, the complication of one patient in Group A was postoperative limb weakness. However, in Group B, one patient developed wound infection after surgery and was successfully treated with antibiotic infusion, and the infection was completely controlled. Three patients underwent hardware removal due to pain. Postoperative limb weakness was observed in four patients. For patients with limb weakness, one-on-one active rehabilitation treatment for patients was advocated, and will follow up regularly for a long time, so that patients can return to normal levels. In summary, in the treatment of PD patients with increased FAA and increased TT-TG distance, the preferred MPFLR + DDFO will obtain better clinical and radiological outcomes.

For the treatment of PD, the most suitable operation should be selected according to the cause of the disease. But whether it is DDFO or TTO, they have high technical requirements and long learning curve (30). The operation and positioning are the key to the success of the operation. For surgeons, especially young and inexperienced doctors, the correct use of anatomical landmarks and precise control of the orthopedic process to achieve the desired effect are often faced with many difficulties (52). In recent years, with the rapid development of imaging and digital medicine, three-dimensional (3D) templates based on CT and patient-specific instruments (PSI), which are embodied by precision and individualization, provide effective means to solve the above problems, and are gradually becoming a research hotspot. Different personalized templates have been designed for cubitus varus deformity and achieved wonderful clinical outcome (53, 54). Lu et al. (55) developed a novel patient-specific template in congenital scoliosis and validated the accuracy and safety. Use of PSI is proposed to lead to improvements in alignment, surgical efficiency, and postoperative patient outcomes, as compared with conventional instrumentation (56). Therefore, future research should focus on the application of PSI systems and 3D printing technology in PD treatment. In theory, this may help to reduce intraoperative errors, better restore the biomechanical characteristics of patients before surgery, improved accuracy of implant size determination and positioning of tibial implant and favourable femoral rotational alignment (57) with PSI systems.

The limitations in this study should also be considered. First, this was a small sample and retrospective study, and more large samples and randomized controlled trials should be conducted in the future. Second, the follow-up time of the study was about 3 years, and the long-term results (e.g., cartilage damage, patellofemoral arthritis, etc.) are not preset and unclear. Third, the two groups did not have contemporaneity, which may lead to selectivity bias. Finally, the proportion of female and male patients was unequal, and the prevalence of PD in female population is higher than that in male population.

This study demonstrated the ability and advantage of MPFLR + DDFO over MPFLR + TTO in treating PD patients with increased FAA and increased TT-TG distance. Regarding clinical relevance, the findings of this study can assist surgeons in surgical decision-making regarding the choice of surgery when treating PD patients with increased FAA and increased TT-TG distance. Based on this study, MPFLR + DDFO can restore a normal mechanical environment of the patellofemoral joint and produce better long-term outcomes.

## Conclusions

Both MPFLR + TTO and MPFLR + DDFO obtained satisfactory clinical and radiological outcomes in the treatment of recurrent PD with increased FAA and excessive TT-TG distance. However, the outcomes of MPFLR + DDFO were better and should be considered a priority. MPFLR + TTO is not necessary for such patients.

## Data Availability

The raw data supporting the conclusions of this article will be made available by the authors, without undue reservation.
